# A Comparative Quantitative Proteomic Analysis of HCMV-Infected Cells Highlights pUL138 as a Multifunctional Protein

**DOI:** 10.3390/molecules25112520

**Published:** 2020-05-28

**Authors:** Yang Li, Weijuan Shang, Gengfu Xiao, Lei-Ke Zhang, Congyi Zheng

**Affiliations:** 1 State Key Laboratory of Virology, College of Life Sciences, Wuhan University, Wuhan 430072, China; 2012202040022@whu.edu.cn; 2 China Center for Type Culture Collection, Wuhan University, Wuhan 430072, China; 3 State Key Laboratory of Virology, Wuhan Institute of Virology, Chinese Academy of Sciences, Wuhan 430071, China; shangweijuan@wh.iov.cn (W.S.); xiaogf@wh.iov.cn (G.X.)

**Keywords:** HCMV, latent infection, UL138, quantitative proteomic

## Abstract

Human cytomegalovirus (HCMV) is a widespread virus that can establish life-long latent infection in large populations. The establishment of latent infection prevents HCMV from being cleared by host cells, and HCMV reactivation from latency can cause severe disease and death in people with immature or compromised immune systems. To establish persistent and latent infection in healthy individuals, HCMV encodes a large array of proteins that can modulate different components and pathways of host cells. It has been reported that pUL138 encoded by the *UL133-UL138* polycistronic locus promotes latent infection in primary CD34+ hematopoietic progenitor cells (HPCs) infected in vitro. In this study, recombinant HCMV Han_UL138del_ was constructed by deleting the *UL138* locus of Han, a clinical HCMV strain. Then, a comparative quantitative proteomic analysis of Han- and Han_UL138del_-infected MRC5 cells was performed to study the effect of pUL138 on host cells in the context of HCMV infection. Our results indicated that, during the early phase of HCMV infection, the innate immune response was differentially activated, while during the late phase of HCMV infection, multiple host proteins were differentially expressed between Han- and Han_UL138del_-infected cells, and these proteins are involved in the oxidation-reduction process, ER to Golgi vesicle-mediated transport, and extracellular matrix organization. Among these proteins, STEAP3, BORCS7, FAM172A, RELL1, and WDR48 were further demonstrated to affect HCMV infection. Our study provides a systematic view of the effect of pUL138 on the host cell proteome and highlights the proposition that multiple biological processes or host factors may be involved in the overall role of the *UL133-UL138* polycistronic locus in HCMV persistence.

## 1. Introduction

Human cytomegalovirus (HCMV) belongs to the Betaherpesvirinae family and is a widespread virus that can establish lifelong latent infection in large populations. In the immunocompetent host, primary infection and reactivation of HCMV are typically asymptomatic [1]. However, in people with immature or compromised immune systems, opportunistic infection or reactivation can cause severe disease and death, particularly in solid-organ transplant patients [2]. Due to the complexity of HCMV-host interactions, the mechanisms by which HCMV regulates the establishment of latency or ensures successful reactivation from latency are currently not clear.

To establish persistent and latent infection in healthy individuals, HCMV encodes a large array of proteins that can modulate different components and pathways of host cells [3]. The genome of HCMV is a linear, double-stranded DNA of ~230 kb in length. All clinical strains of HCMV contain a unique 13- to 15-kb region of the genome named the ULb’ region, which is lost upon serial passage of the virus in fibroblasts, resulting in viruses with higher replicative capacity but more restricted tropism [4,5,6]. Since 20 open reading frames encoded by the ULb’ region are nonessential for viral replication in human fibroblast cells, they have been hypothesized as being important for virus dissemination, latency, or pathogenesis in the human host [7]. 

The *UL133-UL138* polycistronic locus contains genes within the ULb’ region and is important for latency in the experimental CD34^+^ hematopoietic progenitor cell (HPC) model of latency [8,9,10]. Four novel proteins, namely pUL133, pUL135, pUL136, and pUL138, have been shown to be encoded by the *UL133-UL138* locus [11], and it has been reported that pUL138 promotes a latent infection in primary CD34^+^ HPCs infected in vitro. The UL138 protein has been shown to increase cell surface levels of TNFR [12,13], although the significance of these surface alterations to viral infection is not completely understood. 

HCMV infection can profoundly affect the infected cells, resulting in the modulation of cell metabolism, cell cycle, cell death and immune surveillance [14]. These fundamental changes to infected cells may contribute to the establishment of HCMV latency. To study the effect of HCMV infection on host cells, Weekes et al. performed a quantitative temporal proteomic analysis of HCMV-infected HFF cells, in which the modulation of intracellular signaling pathways by HCMV infection was deciphered [15]. Weekes et al. also performed quantitative proteomic analysis of THP-1 cells overexpressing pUL138 in order to monitor which host proteins were regulated by pUL138 and aiming to explore the intracellular signaling pathways that participate in the establishment of HCMV latency mediated by pUL138. They found that the expression of pUL138 resulted in a decrease in MRP1 and that loss of MRP1 and accumulation of the cytotoxic drug vincristine, an MRP1 substrate, decreased the replication of HCMV in latently infected CD14^+^ and CD34^+^ progenitors [16], suggesting that pUL138 may down regulate MRP1 expression to inhibit HCMV replication and thus help to establish persistent infection. 

In this study, we constructed a recombinant HCMV strain based on the clinical strain Han, which was isolated from the urine sample of a Chinese infant with multiple developmental disorders [17]. The recombinant HCMV strain Han_UL138del_ was created by deleting the *UL138* locus of Han. Then, we performed a comparative quantitative proteomic analysis of Han- and Han_UL138del_-infected MRC5 cells. Our results indicated that, during the early phase of HCMV infection, the innate immune response was differentially activated, while during the late phase of HCMV infection, multiple host proteins were differentially expressed between Han- and Han_UL138del_-infected cells. Our study provides a systematic view of the effect of pUL138 on the host cell proteome and highlights the proposition that multiple pUL138-regulating biological processes or host factors may contribute to the overall role of the *UL133-UL138* polycistronic locus in HCMV persistence.

## 2. Results 

### 2.1. Construction of Recombinant HCMV Han_UL138del_

**Figure 1:**
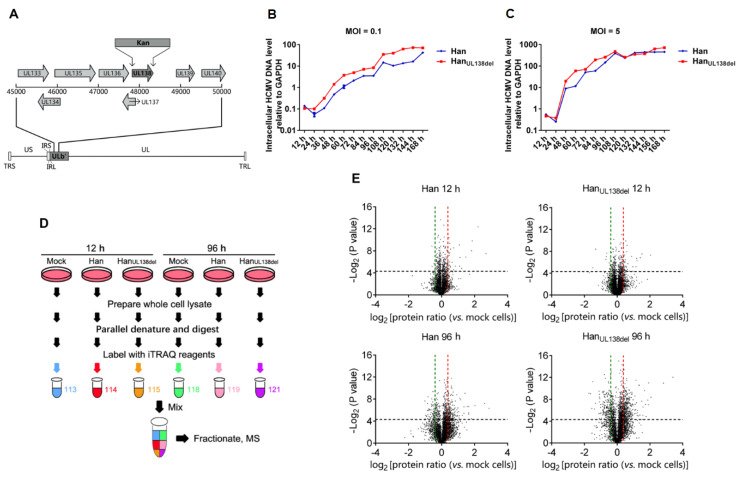
Comparative quantitative proteomic analysis of Han- and Han_UL138del_-infected MRC5 cells. (**A**) Construction of Han_UL138del_-BAC. The ORF of UL138 in the ULb’ region was replaced by the kanamycin resistance cassette. (**B**) Replication kinetics curves of Han and Han_UL138del_ in MRC5 cells infected at an MOI of 0.1. Cells were harvested separately every 12 h for 7 days post infection. Intracellular levels of HCMV genomic DNA were detected with primers for UL44 by quantitative RT-PCR analysis. Three independent biological replicates were performed, and a representative experiment (with two technical replicates) from three biological replicates is shown. Results are shown as mean ± SD. (**C**) Replication kinetics curves of Han and Han_UL138del_ in MRC5 cells infected at an MOI of 5. Three independent biological replicates were performed, and a representative experiment (with two technical replicates) from three biological replicates is shown. Results are shown as mean ± SD. (**D**) Workflow for quantitative proteomic analysis. Cells were infected with Han or Han_UL138del_ (at an MOI of 5) or mock-treated. At 12 h p.i. and 96 h p.i., cells were collected and lysed to extract proteins. Proteins in each sample were subjected to parallel denaturation and digestion. Digested samples were labeled with iTRAQ reagents and mixed together. The mixed peptides were fractionated and analyzed by LC-MS/MS analysis. (**E**) The ratio distribution of quantified proteins.

To study the role of pUL138 in HCMV replication, we reconstituted two strains of HCMV, wild type HCMV and HCMV without pUL138, named Han and Han_UL138del_, respectively (Figure 1A). To monitor the growth kinetics of these two HCMV strains, MRC5 cells were infected with Han or Han_UL138del_ at a multiplicity of infection (MOI) of 0.1 or 5. At different time intervals, the cells were collected, and the level of intracellular HCMV genome was measured by quantitative real-time PCR (RT-PCR). We found that when MRC5 cells were infected with HCMV at an MOI of 0.1, the relative level of intracellular HCMV DNA was lower in Han-infected cells (Figure 1B), while no significant changes were observed in MRC5 cells infected with Han or Han_UL138del_ at an MOI of 5 (Figure 1C). We thus chose an MOI of five as the viral titer to perform quantitative proteomic analysis because the replication efficiencies of these two HCMV strains were similar under these conditions.

### 2.2. Comparative Quantitative Proteomic Analysis of HCMV Han- and Han_UL138del_-Infected MRC5 Cells

To perform quantitative proteomic analysis, MRC5 cells were infected with HCMV Han (MOI of 5) or Han_UL138del_ (MOI of 5) or mock-treated, and the cells were collected at 12 and 96 h post infection (h p.i.) (Figure 1D). Proteins were then extracted and subjected to trypsin digestion, and the digested peptides were labeled with iTRAQ reagents according to the manufacturer’s instructions. Then, equal amounts of the labeled peptides were mixed and subjected to SCX fractionation and LC-MS/MS analysis. Protein identification was performed with ProteinPilot. Three independent biological replicates were performed.

A total of 5119 host proteins were quantified. At 12 h p.i., 48 were upregulated and 29 were downregulated in Han-infected cells, while 79 were upregulated and 58 were downregulated in Han_UL138del_-infected cells. At 96 h p.i., 339 were upregulated and 128 were downregulated in Han- infected cells, while 494 were upregulated and 221 were downregulated in Han_UL138del_-infected cells (Figure 1E, Supplementary Table S1–S3).

**Figure 2:**
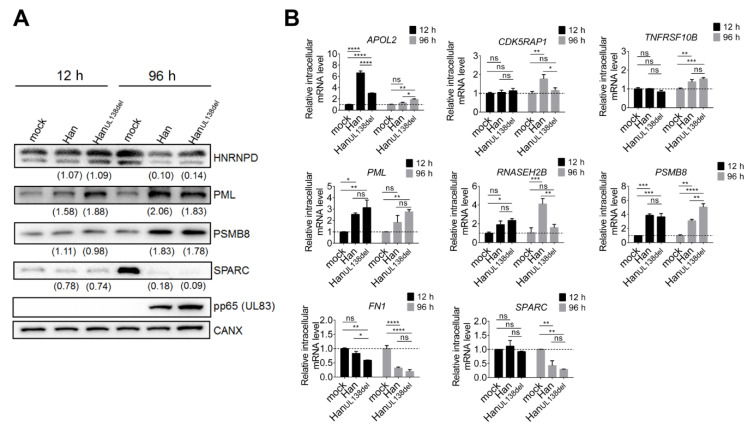
Validation of MS results. (**A**) Western blot analysis of MRC5 cells infected with Han or Han_UL138del_ at an MOI of 5. MRC5 cells were harvested at 12 h p.i. or 96 h p.i., and subjected to Western blot analysis. The intensity of each band was quantitated with Quantity One software and normalized to mock-treated cell band intensity. The numbers with brackets marked below the band represent the relative fold changes. (**B**) Quantitative real-time PCR analysis of selected proteins in cells (MRC5) infected with Han, HanUL138del (at an MOI of 5) or mock-treated. MRC5 cells were harvested at 12 h p.i. or 96 h p.i. and intracellular RNAs were extracted and reverse transcribed to cDNA. The relative mRNA levels of selected proteins were measured by quantitative RT-PCR analysis. Three replicates were performed, and results are shown as mean ± SD. * *p* < 0.05; ** *p* < 0.01; *** *p* < 0.001; **** *p* < 0.0001; ns, no significance.

To validate our MS data, both quantitative RT-PCR and Western blot analysis were performed. Western blot analysis was performed on PML, PSMB8, HNRNPD, RELL1 and SPARC, an exemplary set of proteins, and CANX was selected as the loading control [15]. The intensity of the band was normalized to that in mock-treated cells, and relative fold change were calculated (Figure 2A). We found that, although the fold changes were not identical between the Western blot and MS results, the tendencies were the same. For example, both MS data and WB analysis indicated that PML was upregulated at both time points, while PSMB8 was only upregulated at 96 h p.i. in both HCMV Han- and Han_UL138del_-infected MRC5 cells. WB analysis indicated that HNRPD and SPARC were downregulated at 96 h p.i., while SPARC was slightly downregulated at 12 h p.i. in both HCMV Han- and Han_UL138del_-infected MRC5 cells, and this result was consistent with the MS results.

To further validate our MS results, we also performed quantitative RT-PCR analysis on selected differentially regulated proteins. We found that the mRNA levels of APOL2, CDK5RAP1, TNFRSF10B, PML, RNASEH2B, and PSMB8 were upregulated, while the mRNA levels of FN1 and SPARC were downregulated by infection with HCMV Han and/or Han_UL138del_ (Figure 2B), and these tendencies were consistent with the MS results, suggesting that these proteins were regulated at the mRNA level. Both WB and quantitative RT-PCR analysis supported our MS data.

### 2.3. The Innate Immune Response Was Differentially Regulated between Han and Han_UL138del_

**Figure 3:**
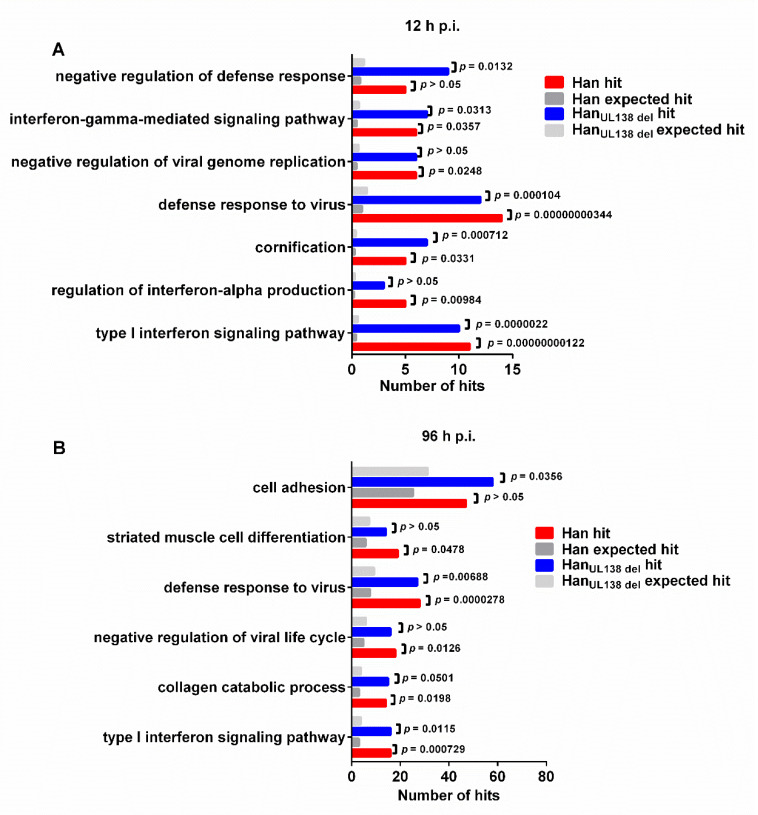
Gene Ontology analysis (biological process) of differentially regulated proteins at 12 h p.i. or 96 h p.i.. Differentially regulated proteins at 12 h p.i. (**A**) or 96 h p.i. (**B**) were subjected to DAVID v6.8. The regulated proteins were grouped based on their roles in biological processes, and a statistical overrepresentation test was performed to determine which biological process was overrepresented by differentially regulated proteins. Only the overrepresented categories are presented here. Expected hits indicate the presumed number of hits in this category.

**Figure 4:**
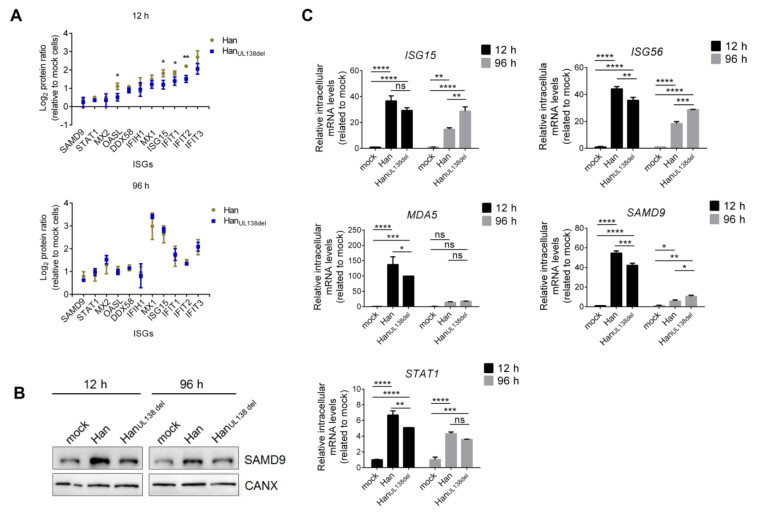
Differential regulation of the innate immune response by Han and Han_UL138del_ at early stage post infection. (**A**) Differential expression of ISGs in Han- and Han_UL138del_-infected MRC5 cells at 12 h p.i. or 96 h p.i.. Y-axis: protein ratios quantified by mass spectrometry (relative to mock-treated cells). (**B**) Western blot analysis of SAMD9 levels in Han- and Han_UL138del_-infected MRC5 cells at 12 h p.i. or 96 h p.i.. SAMD9 was selected as a representative ISG. (**C**) Quantitative RT-PCR analysis of selected ISGs in Han and Han_UL138del_ infected MRC5 cells at 12 h p.i. or 96 h p.i.. ISG15, ISG56, MDA5, SAMD9 and STAT1 were selected as representative ISGs. H: h p.i.. Three replicates were performed, and results are shown as mean ± SD. * *p* < 0.05; ** *p* < 0.01; *** *p* < 0.001; **** *p* < 0.0001; ns, no significance.

At 12 h p.i., 170 proteins were differentially regulated by Han and/or Han_UL138del_, and we then performed gene ontology (GO) analysis on these proteins. We found that the proteins could be grouped into multiple biological processes, including proteins involved in the innate immune response, ubiquitin system, and vesicular transport (Figure 3). No biological processes were specifically regulated by either Han or Han_UL138del_. We found that interferon-stimulated genes (ISGs) constituted a large group of upregulated proteins. Moreover, the upregulation of ISGs by HCMV has also been observed in previous studies [15,18]. However, compared to Han-infected cells, we found that the fold changes of multiple ISGs elevated were higher in Han_UL138del_-infected cells at 12 h p.i. but not at 96 h p.i. (Figure 4A). To confirm this result, we performed WB analysis of SAMD9, a reported ISG with antiviral activity [19]. As shown in Figure 4B, the intracellular protein level of SAMD9 was higher in Han-infected cells than in Han_UL138del_-infected cells at 12 h p.i. (Figure 4B). We also performed quantitative RT-PCR analysis on selected ISGs and found that the mRNA levels of SAMD9, MDA5, ISG15, ISG56, and STAT1 were higher in Han-infected cells than Han_UL138del_-infected cells at 12 h p.i. (Figure 4C). MDA5, ISG15, and ISG56 have been reported that can be upregulated by HCMV infection [15,20], while the HCMV immediate early gene 1 (IE1) can activate STAT1 by re-routing IL-6 signaling [21]..The above results indicated that at the early stage post HCMV infection, both HCMV strains activated the expression of ISGs, while in cells infected with Han, which can express pUL138, a more significant increase in ISG production was observed.

### 2.4. Proteins Differentially Regulated by HCMV Han and Han_UL138del_ at 96 h p.i. Can Affect HCMV Replication

**Figure 5:**
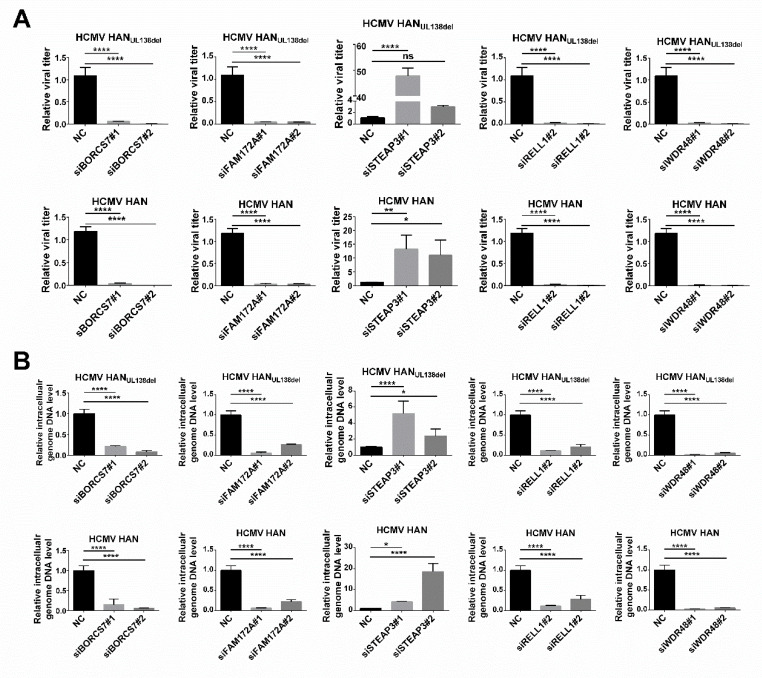
Host proteins differentially regulated by pUL138 at the late stage post HCMV infection can affect HCMV replication. (**A**) The effect of knockdown of BORCS7, FAM172A, RELL1, WDR48 or STEAP3 on HCMV production. MRC5 cells were transfected with siRNA or NC. At 48 h post transfection, the cells were infected with Han or Han_UL138del_ at an MOI of 0.1 or mock-treated. At 5 d post infection, cell culture supernatants were collected, and viral titers in the supernatants were measured. (**B**) The effect of knockdown of BORCS7, FAM172A, RELL1, WDR48 or STEAP3 on intracellular HCMV DNA levels. MRC5 cells were transfected with siRNA or NC. At 48 h post transfection, the cells were infected with Han or Han_UL138del_ at an MOI of 0.1 or mock-treated. At 5 d post infection, the cells were harvested to extract DNA. Intracellular levels of HCMV genomic DNA were detected with primers for UL44 by quantitative RT-PCR analysis.Three replicates were performed, and results are shown as mean ± SD. * *p* < 0.05; ** *p* < 0.01; *** *p* < 0.001; **** *p* < 0.0001; ns, no significance.

Among the 990 host proteins regulated at 96 h p.i., we found that multiple proteins were differentially regulated between HCMV Han and HCMV Han_UL138del_. Next, we explored whether these proteins could affect the replication of HCMV. Briefly, MRC5 cells were transfected with specific siRNAs, and the knockdown efficiency was examined with quantitative RT-PCR (Supplementary Figure S2). Forty-eight hours post siRNA transfection, MRC5 cells were infected with HCMV Han or HCMV Han_UL138del_ at an MOI of five. At 96 h p.i., MRC5 cells were collected, and the intracellular level of HCMV DNA was measured by quantitative RT-PCR. As shown in Figure 5A, knocking down BORCS7, FAM172A, RELL1, and WDR48 reduced intracellular HCMV DNA levels, while knocking down STEAP3 increased intracellular HCMV DNA levels. We also determined the effect of knocking down target proteins on the production of HCMV virions. At 48 h post transfection, MRC5 cells were infected with HCMV Han or HCMV Han_UL138del_ at an MOI of five. At 120 h p.i., the supernatant was collected, and the viral titer was measured. Knocking down BORCS7, FAM172A, RELL1, and WDR48 reduced the production of HCMV virions, while knocking down STEAP3 promoted the production of HCMV virions (Figure 5B). The above results indicated that, at 96 h p.i., multiple host proteins were differentially regulated between Han and Han_UL138del_ in infected MRC5 cells, and among these proteins, BORCS7, FAM172A, RELL1, and WDR48 can promote HCMV infection, while STEAP3 can inhibit HCMV replication.

## 3. Discussion

It has been reported that pUL138 plays an important role in the establishment of latent HCMV infection. To address how pUL138 affects host cell surface protein expression during latent HCMV infection, Weekes et al. performed a quantitative proteomic analysis on myeloid cells with and without pUL138 [16] and found that several differentially regulated proteins may participate in the establishment of latent HCMV infection. However, the effect of pUL138 on the whole cell proteome in the context of HCMV infection is still unknown. To this end, we constructed an HCMV strain without UL138 (HCMV Han_UL138del_), derived from the HCMV clinical strain Han, and performed comparative quantitative proteomic analysis of MRC5 cells infected with HCMV Han and HCMV Han_UL138del_. A total of 5119 proteins were quantified at both 12 and 96 h p.i., and hundreds of differentially expressed proteins were identified (Supplementary Table S1–S3 and Figure S1). Considering that quantification with mixed protein sources from host and virus might cause errors, we thus employed Western blot and quantitative RT-PCR analysis on selected proteins to verify our MS results. GO analysis indicated that, at 12 h p.i., the early stage post HCMV infection, proteins involved in innate immune response, ubiquitin system, and vesicular transport were differentially regulated by both HCMV Han and HCMV Han_UL138del_ infection. Further examination of protein ratio distributions indicated that the levels of regulated ISGs were higher in HCMV Han-infected MRC5 cells than in HCMV Han_UL138del_-infected MRC5 cells, suggesting that the production of ISGs was more highly activated after HCMV Han infection. It has been reported that pUL138 can be expressed at 6 h p.i. in HCMV-infected cells [22], and we speculate that the expression of pUL138 at this time point might activate the production of ISGs and subsequently suppress HCMV replication to help establish latent infection. Indeed, we found that HCMV replication was lower in the presence of pUL138 when the cells were infected with a low MOI of HCMV (Figure 1B).

As HCMV started to replicate and more viral proteins were expressed, a more significant effect on the host proteome was observed in HCMV infected cells at 96 h p.i., the late stage post HCMV infection. However, the expression levels of ISGs were comparable between Han- and Han_UL138del_-infected cells at this time point, which may be because other viral proteins that have been reported to affect the innate immune response were also expressed. More differentially regulated proteins were identified at 96 h p.i., and we found that multiple proteins were differentially regulated between Han- and Han_UL138del_-infected cells. We then performed functional analysis of selected proteins and found that BORCS7, FAM172A, STEAP, RELL1, and WDR48 could affect HCMV replication. 

Among these proteins, RELL1 is a membrane protein that can induce cellular death in HEK 293 epithelial cells [23]. Here, we found that intracellular level of RELL1 in Han-infected cells was lower than that in Han_UL138del_ cells, suggesting that pUL138 may downregulate RELL1 expression during HCMV infection. A previous quantitative proteomic analysis also indicated that RELL1 was downregulated in pUL138-expressing THP-1 cells [16]. Thereafter, we explored whether RELL1 could affect HCMV replication and found that knocking down RELL1 could decrease both HCMV replication and the virus titer (Figure 5), suggesting that RELL1 can promote HCMV replication. The downregulation of RELL1 by pUL138 during HCMV infection may inhibit HCMV replication, thus favoring the establishment of latent infection. 

WDR48 is a regulator of deubiquitinating complexes [24]. It has been reported that herpes virus saimiri Tip protein can interact with WDR48 to down regulate T cell receptor (TCR) and CD4 surface expression. Moreover, herpes viruses may employ this mechanism to deregulate lymphocyte receptor expression to disarm host immune control [25]. Here, we found that WDR48 was downregulated in Han-infected cells but not in Han_UL138del_-infected cells, suggesting that pUL138 may decrease intracellular WDR48 levels. We further found that knocking down WDR48 impaired HCMV replication, suggesting that pUL138 may downregulate this protein to inhibit HCMV replication. We also found that the protein level of FAM172A in HCMV Han-infected cells was lower than that in HCMV Han_UL138del_-infected cells, and knocking down FAM172A decreased HCMV replication, while the intracellular protein level of STEAP3 in HCMV Han-infected cells was higher than that in HCMV Han_UL138del_-infected cells, and knocking down STEAP3 increased HCMV replication, suggesting that pUL138 may upregulate STEAP3 to inhibit HCMV replication. 

In conclusion, our study provides a comprehensive view of the effects of pUL138 on the host cell proteome in the context of HCMV infection. Our results indicated that, during the early phase of HCMV infection, the innate immune response was differentially activated as a result of pUL138 expression. During the late phase of HCMV infection, STEAP3, BORCS7, FAM172A, RELL1 and WDR48 were differentially expressed between Han- and Han_UL138del_-infected cells, and further functional studies indicated that all of these proteins could affect HCMV infection. Our study provides a systematic view of the effect of pUL138 on the host cell proteome and highlights the proposition that multiple biological processes or host factors may contribute to the overall role of the *UL133-UL138* polycistronic locus in HCMV persistence.

## 4. Materials and Methods

### 4.1. Cells

Human fetal lung fibroblasts (MRC5), HEK 293T cells and HeLa cells were obtained from CCTCC. MRC5 cells were cultured in minimum Eagle’s medium (MEM) supplemented with 10% fetal bovine serum (FBS), 100 U/mL penicillin, and 100 μg/mL streptomycin at 37 °C with 5% CO_2_. HEK293T and HeLa cells were cultured in Dulbecco’s modified Eagle’s medium (DMEM) supplemented with 10% FBS, 100 U/mL penicillin, and 100 μg/mL streptomycin at 37 °C with 5% CO_2_.

### 4.2. Viruses 

HCMV Han DNA was a gift from Dr. Minhua Luo (Wuhan Institute of Virology, CAS) [17]. HCMV Han was cloned into a bacterial artificial chromosome (BAC) that contains green fluorescent protein (GFP) to obtain Han-BAC DNA. Han-BAC DNA was amplified in recombinogenic *Escherichia coli* strain EL350. Briefly, Han BAC DNA was electroporated into *E. coli* EL350 using a Gene Pulser Xcell (Bio-Rad, Hercules, CA, USA) with 0.1 cm-gap cuvette at 1600 V. An EL350 clone containing Han BAC was selected on LB agar plates supplemented with 12.5 µg/mL chloramphenicol and then amplified in LB liquid medium supplemented with 12.5 µg/mL chloramphenicol. Then, EL350 were collected, and Han-BAC DNA was purified with NucleoBond Xtra Midi (Macherey-Nagel, Düren, Germany).

To obtain Han-BAC DNA without UL138, a DNA segment carrying a kanamycin resistance cassette flanked by sequences homologous to sequences flanking *UL138* in the Han genome was amplified from the pGBKT7 plasmid and electroporated into *E. coli* EL350, in which *UL138* was replaced by *kan* via homologous recombination. An EL350 clone containing Han-BAC_UL138del_ DNA was selected on LB agar plates supplemented with 12.5 µg/mL chloramphenicol and 30 µg/mL kanamycin. Han-BAC_UL138del_ DNA was extracted as described above. 

To reconstitute the viruses, BAC DNA and the pcDNA3.1-UL82 plasmid were electroporated into MRC5 cells with a 0.4-cm-gap cuvette at 260 V. HCMV virions were collected after MRC5 cells were subjected to three freeze-thaw cycles. Cell debris was removed from the suspension by centrifugation at 2000 rpm for 10 min at 4 °C. Virions were concentrated by ultracentrifugation through a 35% (wt/vol) sucrose cushion at 25,000 rpm (~77,000× g) in an SW32 rotor (Beckman Coulter, USA) for 2 h at 4 °C. Virus particles were resuspended in MEM with 10% FBS and 1% DMSO and stored at –80 °C.

#### 4.2.1. Viral DNA Purification

MRC5 cells were seeded onto 12-well plates at 1 × 10^5^ cells per well and infected with HCMV Han or Han_UL138del_ at an MOI of 0.1 or 5 and were harvested using cell scarpers at different time intervals and stored at −80 °C. All samples were lysed with Buffer A in the TIANamp Genomic DNA Kit (Tiangen, Beijing, China), and DNA was purified following the manufacturer’s protocol.

#### 4.2.2. RNA Purification

Cells were lysed in RNAiso Plus (Takara, Dalian, China) to extract total RNA. Then, RNA was reverse transcribed to cDNA using SYBR^®^ Premix EX Taq™ II (Takara, Dalian, China) following the manufacturer’s protocol. The relative mRNA levels of selected proteins were measured by quantitative RT-PCR analysis. 

#### 4.2.3. Quantitative Real-Time PCR 

Quantitative RT-PCR was performed in a total volume of 20 μl using TB Green Premix Ex Taq™ II (Takara, Dalian, China) on an ABI StepOnePlus™ Real-Time PCR System (Applied Biosystems, Foster City, CA) using the following protocol: 50 °C for 2 min and 95 °C for 2 min, then 40 cycles of 95 °C for 15 s and 60 °C for 1 min, ending with the melting curve stage.

#### 4.2.4. Protein Extraction

HCMV-infected or mock-treated MRC5 cells were collected and washed with prechilled PBS three times. Cell pellets were resuspended in lysis buffer (8 M urea/0.2 M Tris, pH 8.0) and subjected to sonication. After centrifugation, supernatants containing the extracted proteins were collected, and protein abundance was measured with a bicinchoninic acid (BCA) assay. Extracted proteins were reduced with 10 mM DTT at 56 °C for 30 min, alkylated with 40 mM iodoacetamide in the dark for 30 min, and left in light at room temperature for 2 h. Proteins were digested with trypsin (Promega) at a ratio of 1:50 (trypsin/protein w/w) overnight at 37 °C, and the digested peptides were desalted with SepPak C18 cartridge (Waters) and dried by Speed Vac (Thermo).

#### 4.2.5. iTRAQ Labeling and LC-MS/MS Analysis 

For iTRAQ labeling, 100 μg of peptides from HCMV Han-, HCMV Han_UL138del_- or mock-infected cells were labeled with different iTRAQ reagents according to the manufacturer’s instructions (SCIEX), and equal amounts of labeled peptides were mixed and desalted. The mixed peptides were fractionated into 8 fractions using strong cation exchange (SCX) as previously described [26]. The fractionated peptides were dried by Speed Vac and stored at −80 °C.

LC-MS/MS analysis using NanoLC-Ultra 1D plus (Eksigent) was performed on a quadrupole-TOF LC/MS/MS mass spectrometer (TripleTOF 5600+, SCIEX) equipped with a nanospray source. Peptides dissolved in loading buffer (2% ACN/97.9% H_2_O/0.1% formic acid) were first loaded onto a C18 trap column (5 µm, 5 × 0.3 mm, Agilent Technologies) and then eluted into a C18 analytical column (75 μm × 150 mm, 3 μm particle size, 100 Å pore size, Eksigent). Mobile phase A (3% DMSO, 96.9% H2O, 0.1% formic acid) and mobile phase B (3% DMSO, 96.9% ACN, 0.1% formic acid) were used to establish a 100 min gradient, which comprised of: 0 min in 5% B, 65 min of 5–23% B, 20 min of 23–52% B, 1 min of 52–80% B, the gradient was maintained in 80% B for 4 min, followed by 0.1 min of 80–85% B, and a final step in 5% B for 10 min. The constant flow rate was set at 300 nL/min. For MS/MS analysis, each scan cycle consisted of one full-scan mass spectrum (with m/z ranging from 350 to 1500 and charge states from 2 to 5) followed by 20 MS/MS events. The threshold count was set to 120 to activate MS/MS accumulation, and former target ion exclusion was set for 18 s. Mass spectra were extracted by Peakview v2.0 (SCIEX).

### 4.3. Experimental Design and Statistical Rationale

Three independent biological replicates were performed, and peptides from three biological replicates were analyzed by LC-MS/MS independently. The mass spectrometry proteomics data have been deposited to the ProteomeXchange Consortium [27] via the PRIDE partner repository with the dataset identifier <PXD015931>.

MS spectra were subjected to ProteinPilot v5.0.1 (SCIEX) analysis for peptide identification and quantification. A concatenated database (n = 20355) containing the HCMV protein sequence and the UniProt_Human database (2016/05) was used. Search parameters were as follows: sample type: iTRAQ 8plex (peptide labeled); cysteine alkylation: iodoacetamide; digestion: trypsin; miss cleavage tolerance: 2; fixed modification: carbamidomethyl Cys; variable modification: none; MS1 initial mass error tolerance value: 0.05 Dalton; MS2 initial mass error tolerance value: 0.1 Dalton; Instrument: TripleTOF 5600. The false discovery rate (FDR) analysis in ProteinPilot uses the “decoy database searching” strategy, and in this study the FDRs of the ProteinPilot search results were all set as lower than 1% at the protein level.

For protein quantification, all quantified peptides were exported, and only the peptides with a confidence score >95% were kept for further analysis. In each replicate, the protein ratio was calculated by weighted average ratios of its peptides, with peptide intensity as the weight. The protein ratio values used for bioinformatics analysis were the weighted averages of the three biological replicates, while the *p* value for the protein ratio was calculated and further corrected with multiple Bonferroni correction (Supplementary Table S1). All quantified proteins presented had at least two quantified peptides. The cutoff for differentially regulated proteins was set as described in a previous study [28,29]. Briefly, the Gaussian distribution of protein ratios was analyzed, and values deviating from the mean of the normally distributed data by 3.3 standard deviations were considered as cutoff values. Only proteins that met the following two criteria were considered differentially regulated: (1) with ratios > upregulated or < downregulated cutoff values, and (2) with corrected protein ratio *p* value < 0.05.

### 4.4. Gene Ontology (GO) Analysis

To perform GO analysis, differentially regulated proteins were submitted to DAVID 6.8 [30]. Proteins were classified into different categories, and a statistical over-representation test was performed. *p* values were assessed with a binomial test and corrected for multiple testing using a Bonferroni procedure. Only categories with a *p* value < 0.05 were considered as over- or under- represented.

### 4.5. Western Blotting

Cells were lysed in RIPA lysis buffer (Beyotime, Shanghai, China) or 1X SDS-loading buffer with PMSF. Protein lysates were fractionated by sodium dodecyl sulfate-polyacrylamide gel electrophoresis in 10 to 15% gels and transferred to 0.2 µm PVDF membranes in semi-dry transfer system (Bio-Rad, Hercules, CA, USA). Membranes were blocked in Tris-buffered saline with 0.1% Tween-20 (TBST) containing 5% nonfat dry milk for 2 h at room temperature. Membranes were incubated with primary antibodies in TBST overnight at 4 °C. After washing with TBST (three times, 20 min each), membranes were incubated with horseradish peroxidase-labeled secondary antibodies in TBST containing 5% nonfat dry milk for 1 h at room temperature. The bands were developed with enhanced chemiluminescence (ECL) (Millipore, Billerica, MA) and visualized by the ChemiDoc MP Imaging System (Bio-Rad, Hercules, CA, USA). The following antibodies (Abs) were used: anti-HNRNPD polyclonal Ab (ProteinTech, Wuhan, China), anti-PML polyclonal Ab (ProteinTech, Wuhan, China), anti-PSMB8 polyclonal Ab, anti-SPARC polyclonal Ab, anti-pp65 monoclonal Ab (Santa Cruz Biotechnology, Dallas, TX), and anti-CANX polyclonal Ab (ABclonal, Wuhan, China).

## Supplementary Materials

The following Supplementary Materials can be found online: Table S1. Ratio of protein quantified in HCMV infected cells; Table S2. Protein ratios of Han vs. HanUL138del at 12 and 96 h p.i.; Table S3. Protein ratios of late infection (96 h p.i.) vs. early infection (12 h p.i.) in Han and HanUL138del infected cells; Figure S1. The ratio distribution of quantified proteins; Figure S2. Knockdown efficiency of target gene-specific siRNAs.
